# Pilot Study: Short Term Impact of Radiation Therapy on Bone Mineral Density and Bone Metabolism

**DOI:** 10.1007/s00223-023-01149-1

**Published:** 2023-11-01

**Authors:** Quirina C. B. S. Thio, Olivier D. R. van Wulfften Palthe, Jos A. M. Bramer, Thomas F. DeLaney, Miriam A. Bredella, David W. Dempster, Hua Zhou, Francis J. Hornicek, Yen-Lin E. Chen, Joseph H. Schwab

**Affiliations:** 1 Department of Orthopedic Surgery, Academic University Medical Center, University of Amsterdam, Amsterdam, The Netherlands; 2 Department of Orthopedic Surgery, Massachusetts General Hospital, Harvard Medical School, Boston, MA USA; 3 Department of Radiation Oncology, Massachusetts General Hospital, Harvard Medical School, Boston, MA USA; 4 Department of Radiology, Massachusetts General Hospital, Harvard Medical School, Boston, MA USA; 5 Department of Pathology and Cell Biology, Columbia University, New York, NY USA; 6 Regional Bone Center, Helen Hayes Hospital, West Haverstraw, New York, USA; 7 Department of Orthopedic Surgery, UCLA Health, Los Angeles, CA USA; 8 Massachusetts General Hospital, Room 3.946, Yawkey Building, 55 Fruit Street, Boston, MA 02114 USA

**Keywords:** Radiation therapy, Bone, Chordoma, Chondrosarcoma, Bone histomorphometry

## Abstract

Despite the risk of complications, high dose radiation therapy is increasingly utilized in the management of selected bone malignancies. In this study, we investigate the impact of moderate to high dose radiation (over 50 Gy) on bone metabolism and structure. Between 2015 and 2018, patients with a primary malignant bone tumor of the sacrum that were either treated with high dose definitive radiation only or a combination of moderate to high dose radiation and surgery were prospectively enrolled at a single institution. Quantitative CTs were performed before and after radiation to determine changes in volumetric bone mineral density (BMD) of the irradiated and non-irradiated spine. Bone histomorphometry was performed on biopsies of the irradiated sacrum and the non-irradiated iliac crest of surgical patients using a quadruple tetracycline labeling protocol. In total, 9 patients were enrolled. Two patients received radiation only (median dose 78.3 Gy) and 7 patients received a combination of preoperative radiation (median dose 50.4 Gy), followed by surgery. Volumetric BMD of the non-irradiated lumbar spine did not change significantly after radiation, while the BMD of the irradiated sacrum did (pre-radiation median: 108.0 mg/cm^3^ (IQR 91.8–167.1); post-radiation median: 75.3 mg/cm^3^ (IQR 57.1–110.2); p = 0.010). The cancellous bone of the non-irradiated iliac crest had a stable bone formation rate, while the irradiated sacrum showed a significant decrease in bone formation rate [pre-radiation median: 0.005 mm^3^/mm^2^/year (IQR 0.003–0.009), post-radiation median: 0.001 mm^3^/mm^2^/year (IQR 0.001–0.001); p = 0.043]. Similar effects were seen in the cancellous and endocortical envelopes. This pilot study shows a decrease of volumetric BMD and bone formation rate after high-dose radiation therapy. Further studies with larger cohorts and other endpoints are needed to get more insight into the effect of radiation on bone. *Level of evidence: IV.*

## Introduction

### Background

Radiation therapy (RT) plays an important role in the management of selected bone malignancies. Combined with surgical resection, RT was shown to significantly improve outcome in historically difficult to treat tumors, such as chordoma [1–4]. However, the rate of insufficiency fractures was found to be relatively high after RT and surgery (60%) or definitive RT alone (33%) [5, 6]. Even at relatively low doses of RT [30 to 40 Gray (Gy)], skeletal complications such as radiation-induced osteoporosis, insufficiency fractures, physeal arrest, and non-union are common [7–11]. These detrimental effects of RT increase proportionally with the dose delivered [12]. Despite the risk of complications, high-dose RT is becoming increasingly utilized because of its improvement in local tumor control. For example, the combination of surgery and adjuvant RT doses of greater than 70 Gy achieves meaningful control of locally aggressive tumors, that otherwise have a very high rate of recurrence [2].

Such is the case for sacral malignant bone tumors. These rare tumors, mostly chordoma, can be challenging to treat with surgery alone and have a very high rate of recurrence after surgery alone [13]. They are often treated with moderate to high dose RT, with or without surgery. The sacrum is one of the bones maximally affected by post-radiation complications [6] In the current study we investigated the impact of moderate to high dose RT (over 50 Gy) on BMD and bone metabolism, as much remains unknown about the biological mechanisms and structural changes that occur in human bone in response to high dose of RT. To minimize variability in bone properties, we chose to focus this study on the sacrum. In a previous retrospective computed tomography (CT) study, trabecular bone mineral density (BMD) was found to be decreased in the irradiated bone after high-dose radiation, while it remained stable in the non-irradiated bone [14]. In this pilot study we aimed to prospectively confirm these findings and additionally to investigate other effects of high-dose RT on bone by analyzing bone biopsies of patients treated for a malignant tumor of the sacrum.

### Objectives

The primary objective of this study was to measure the effect of RT on volumetric BMD in the adult sacrum using quantitative CT (QCT) in patients undergoing treatment with combination preoperative RT and surgery or RT alone.

The secondary objectives were to characterize the effect of RT on the dynamic (e.g. mineral apposition rate, bone formation rate, and mineralization lag time) and static parameters of bone turnover (e.g., eroded surface, osteoid surface and volume) by tetracycline quadruple labeling and bone histomorphometry.

## Materials and Methods

### Study Design

This prospective pilot study was carried out according to the STrengthening the Reporting of OBservational studies in Epidemiology (STROBE) statement [15] and was approved by our Institutional Review Board.

### Participants

Inclusion criteria for this study were: (1) histologically confirmed primary malignant bone tumor in the sacrum for which surgery and radiation or radiation alone was planned, (2) age 18 years or older, (3) normal organ and marrow function (for surgical arm). Normal organ and marrow function was defined as: total bilirubin within normal institutional limits, AST (SGOT)/ALT (SGPT) < 2.5 × institutional upper limit of normal, and creatinine within normal institutional limits or creatinine clearance > 60 mL/min/1.73 m^2^ for subjects with creatinine levels above institutional normal limit, (4) ability to understand and willingness to sign a written informed consent.

As tetracyclines and ionizing radiation are harmful to the developing human fetus, women of child-bearing potential had to agree to use adequate contraception (hormonal or barrier method of birth control; abstinence) prior to study entry and until after the last study-related CT scan.

Exclusion criteria for this study were: (1) history of surgery, chemotherapy, or RT of the sacrum prior to the study, (2) history of allergic reactions attributed to compounds of similar chemical or biologic composition to tetracyclines (for surgical arm), (3) pregnancy or nursing, (4) uncontrolled illness including, but not limited to ongoing or active infection, symptomatic congestive heart failure, unstable angina pectoris, cardiac arrhythmia, or psychiatric illness/social situations that would limit compliance with study requirements.

### Treatment Protocol

**Fig. 1:**
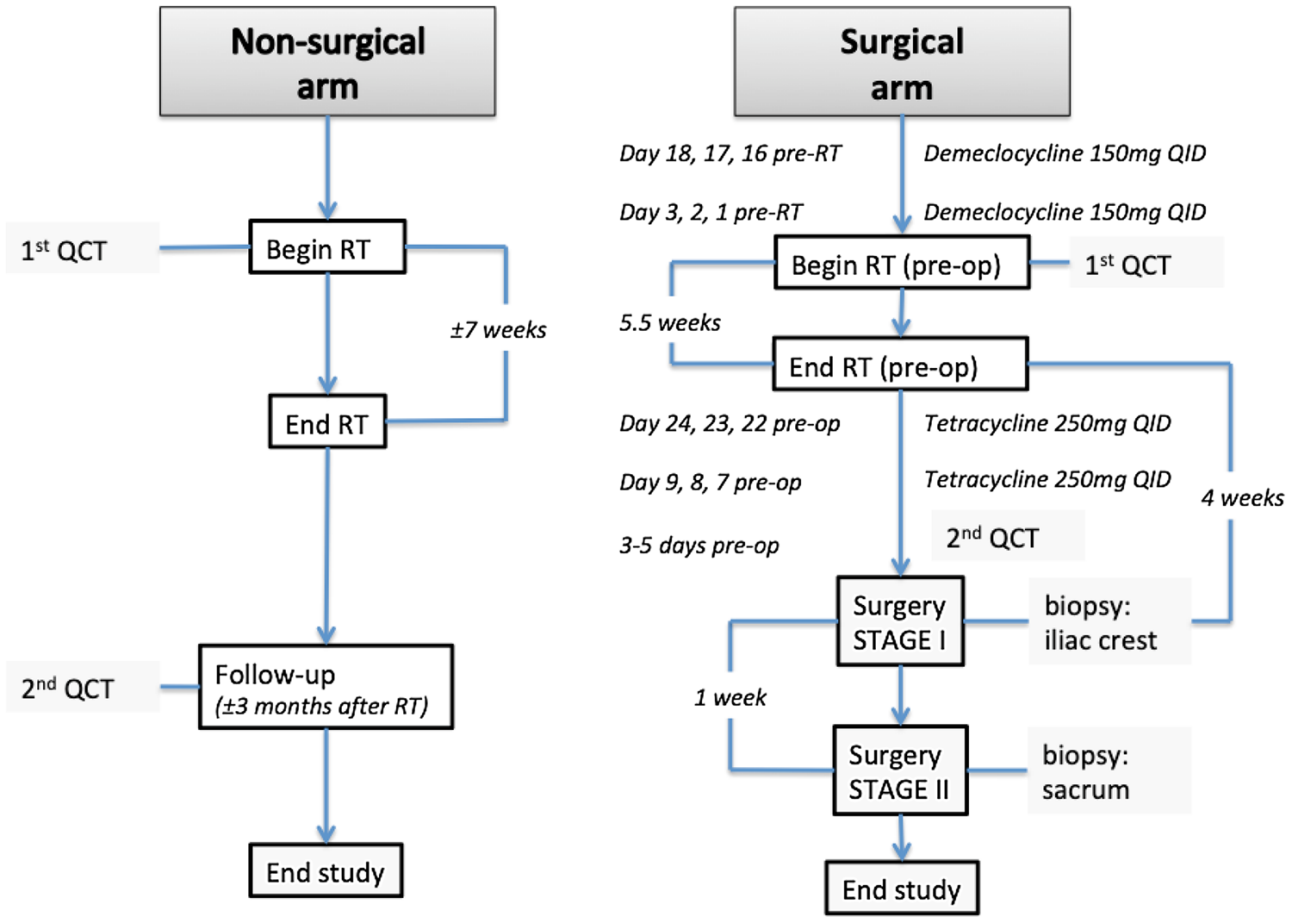
Timeline for both the non-surgical and the surgical arm

**Fig. 2:**
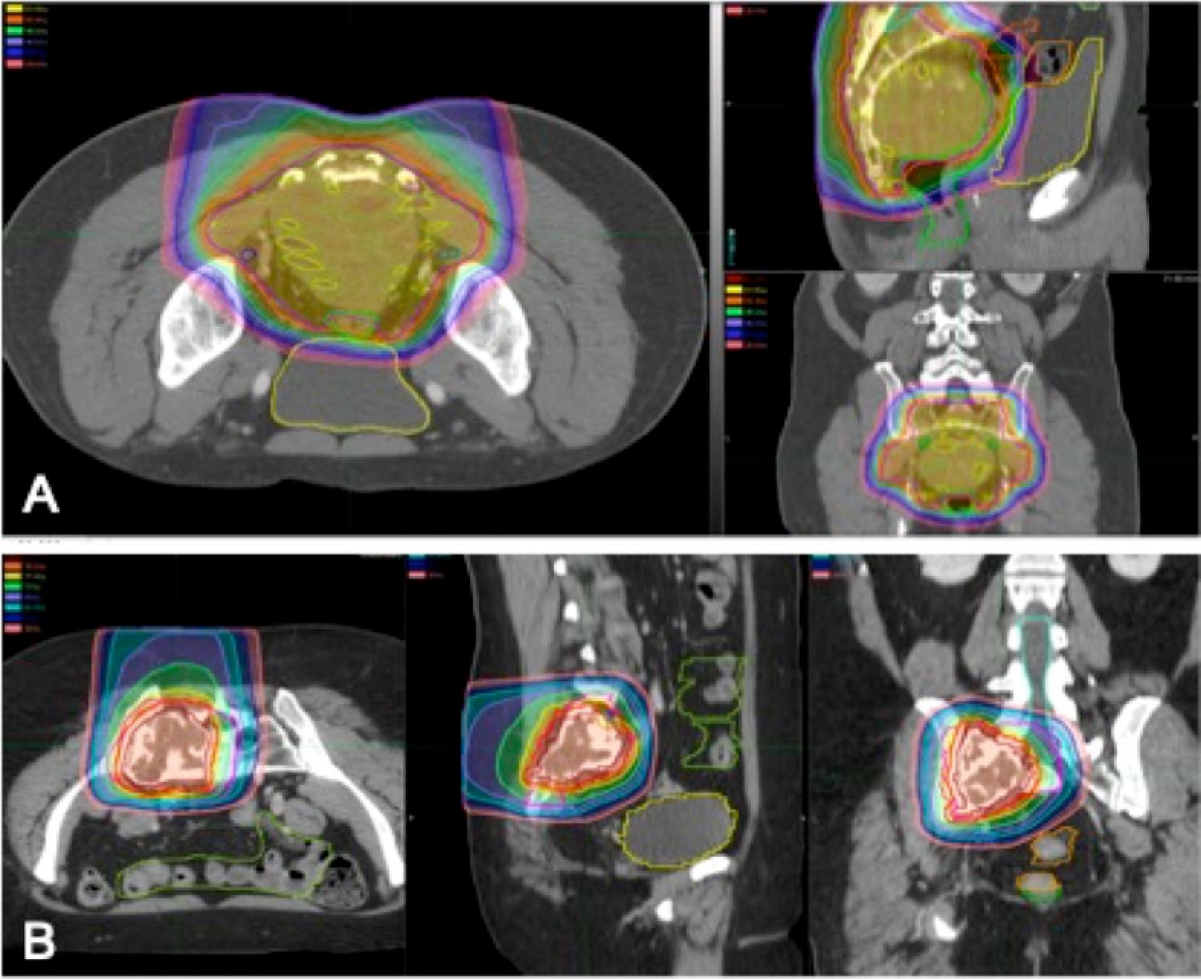
Radiation field of **A** preoperative 50.4 Gy RT for sacral chordoma followed by resection and **B** definitive RT for sacral chondrosarcoma

Patients were enrolled between September 2015 and April 2018 at a single tertiary referral center, prior to the start of treatment for a primary malignant sacral tumor. According to their treatment plan (high-dose RT alone, or a combination of preoperative high-dose RT and surgery followed by additional postoperative RT), determined by the treating physicians and the patients themselves, patients were either enrolled in the non-surgical arm or the surgical arm (Fig. 1). The non-surgical arm consisted of a period of seven weeks of RT (77.4–79.2 Gy), while the surgical arm received pre-operative RT (50.4 Gy in all patients) and post-operative RT (19.8–27 Gy depending on final margins). RT consisted of a combination of protons and/or photons. Radiation treatment clinical target volume (CTV) consisted of a gross target volume (GTV) including MRI and CT extent of gross tumor plus expansion margins to include vertebral body and spinal canal extension of one level above and below to account for potential microscopic extension through the dorsal venous plexus or spinal canal, plus 2 to 3 cm of margin into involved muscles such as erector spinae, gluteus muscles, and/or piriformis muscles (Fig. 2). All subjects underwent two QCTs, and for the surgical arm two biopsies. All patients in the non-surgical arm were followed until after their last radiation treatment, while all patients in the surgical arm were followed until after their last surgery date.

### Quantitative CT

All subjects underwent QCT of the L1 and L2 vertebra and the sacrum by using a second-generation dual-source 128-row multidetector CT scanner (Somatom Definition Flash; Siemens Medical Solutions, Forchheim, Germany). Subjects were positioned on a QCTPro calibration phantom (Mindways Software, Inc., Austin, TX). Helical scans were performed at 80 and 140 kV by using 210 and 80 mA, respectively. Other scanning parameters included 1-s gantry rotation time, 0.9:1 pitch, and 64 × 0.6-mm detector configuration with double z-sampling. The images were reconstructed at 2-mm section thickness and 2-mm section interval by using the I31f reconstruction kernel with a sinogram-affirmed iterative reconstruction (SAFIRE; Siemens Healthcare). Single-energy volumetric BMD of the lumbar spine and sacrum was assessed using the 140-kV data set. Three-dimensional reconstructive analysis was performed by using QCT PRO software version 5.1 (Mindways Software, Inc., Austin, TX).

For patients in the surgical arm, QCT was performed prior to the start of pre-operative RT and after completion of pre-operative RT (50.4 Gy). For patients in the non-surgical arm, QCT scan was performed prior to the start of their RT and at approximately 3 months after RT.

### Bone Histomorphometry

In the surgical arm, patients self-administered two cycles of the bone-labeling drug demeclocycline (150 mg, four times a day by mouth) 18 days before starting RT, using a standard 3 days-on, 12 days-off, 3 days-on regimen. Twenty-four days before surgery, shortly after completion of 50.4 Gy preoperative RT, patients self-administered two cycles of the second bone-labeling drug, tetracycline (250 mg, four times a day by mouth), also using a standard 3 days-on, 12 days-off, 3 days-on regimen. The use of the quadruple labeling technique allowed longitudinal assessment of the dynamic parameters of bone formation before and after RT in a single biopsy sample [16, 17]. During the two-stage surgery, biopsies were taken: a biopsy from the non-irradiated iliac crest during the first stage and a biopsy from the radiated sacrum during the second stage.

Samples were prepared and analyzed as previously described [16, 17]. Briefly, biopsy samples were fixed in 70% ETOH, dehydrated, and embedded in methyl methacrylate. Thin sections were cut at 7 μm prior to staining with Goldner's trichrome and toluidine blue for analysis of static parameters, and at 20 μm, unstained, for analysis of dynamic parameters. All histomorphometric analysis was performed by the same individual (HZ) using OsteoMeasure software (OsteoMetrics Inc., Decatur, GA). All indices were calculated and according to the recommendations of the ASBMR Nomenclature Committee [18]. One patient in the surgical arm did not take the second two cycles of tetracycline, which made it impossible to obtain post-treatment bone formation parameters by bone histomorphometry.

### Statistical Analyses

Berthold and Haras published a range of normal reference values for the trabecular bone mineral density measured by quantitative CT of the lumbar spine in young adults [19]. In healthy males and females the mean trabecular bone mineral density was 150 mg/mL with a standard deviation of 20 mg/mL at the start of puberty. Assuming that in our population this is at least 1 standard deviation less that of a normal young adult, we get a reference estimate of 130 mg/dL at the start of treatment in all study patients. An ante-hoc power calculation determined that a sample of 8 patients would provide 89% of statistical power (alpha 0.05) to detect a difference of 20 mg/cm^3^ assuming a standard deviation of the change is 15 mg/cm^3^.

Wilcoxon signed-rank tests were performed to assess the difference between variables before and after radiation. A two-sided p value of < 0.05 was considered significant. All statistical analyses were performed using Stata Version 13.0 (Stata Corp, College Station, TX, USA).

## Results

### Patient Characteristics

Between September 2015 and April 2018, 31 patients were treated for a sacral tumor at our institute. Nine of these patients consented to participate in the study, of which two chose to be treated with RT only and seven with a combination of RT and surgery. The baseline characteristics of the study cohort are listed in Table 1. Six patients (67%) were treated for a chordoma while three patients (33%) were treated for a chondrosarcoma.

**Table 1:** Baseline characteristics

Variable	Non-surgical arm (n = 2)	Surgical arm (n = 7)
	%/Median (IQR)	%/Median (IQR)
Age (years)	63 (53–73)	55 (42–64)
Female sex	50%	14.30%
BMI (kg/m^2^)	26.3 (23.1–29.5)	27.1 (26.58–31)
Primary tumor type		
Chordoma	50%	71.40%
Chondrosarcoma	50%	28.60%
Radiation dose, range (Gy)	77.4–79.2*	50.4*
Highest level of tumor		
S1	1 (50%)	1 (14%)
S2	1 (50%)	2 (29%)
S3		3 (43%)
S4		1 (14%)

*Total of photons and protons

### Bone Mineral Density

**Fig. 3:**
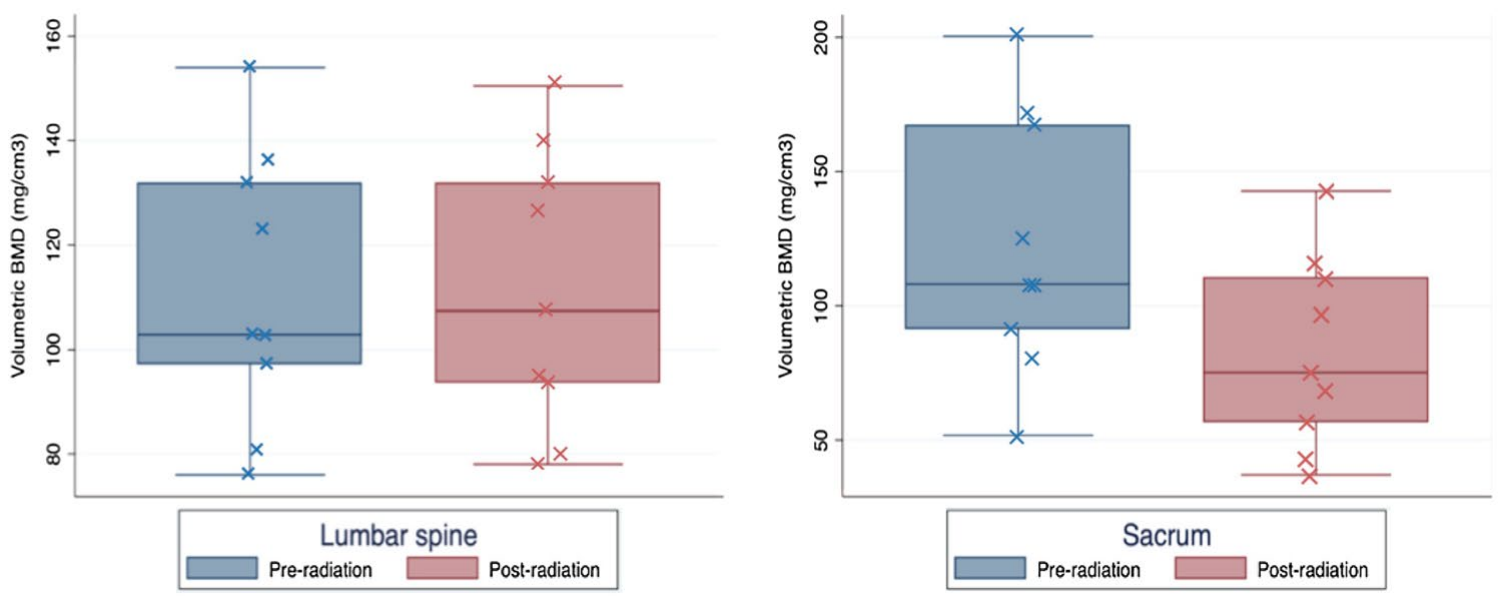
Boxplots showing the volumetric bone mineral density (BMD) of the non-irradiated lumbar spine and the irradiated sacrum before and after RT. The difference in BMD was not significant in the lumbar spine (p = 0.767) and significant in the sacrum (p = 0.010)

Figure 3 shows the boxplots of volumetric BMD. In the lumbar spine (L1 and L2), outside of the radiation field, the median BMD prior to radiation was 102.9 mg/cm^3^ (IQR 97.2–131.8), and after radiation was 107.4 mg/cm^3^ (IQR 93.7–131.6). The difference was not significant (p = 0.767). Within the radiation field, there was a significant decrease in volumetric BMD of the sacrum, median BMD prior to radiation 108.0 mg/cm^3^ (IQR 91.8–167.1) vs. 75.3 mg/cm^3^ (IQR 57.1–110.2) after radiation, p = 0.010.

### Bone Histomorphometry

**Fig. 4:**
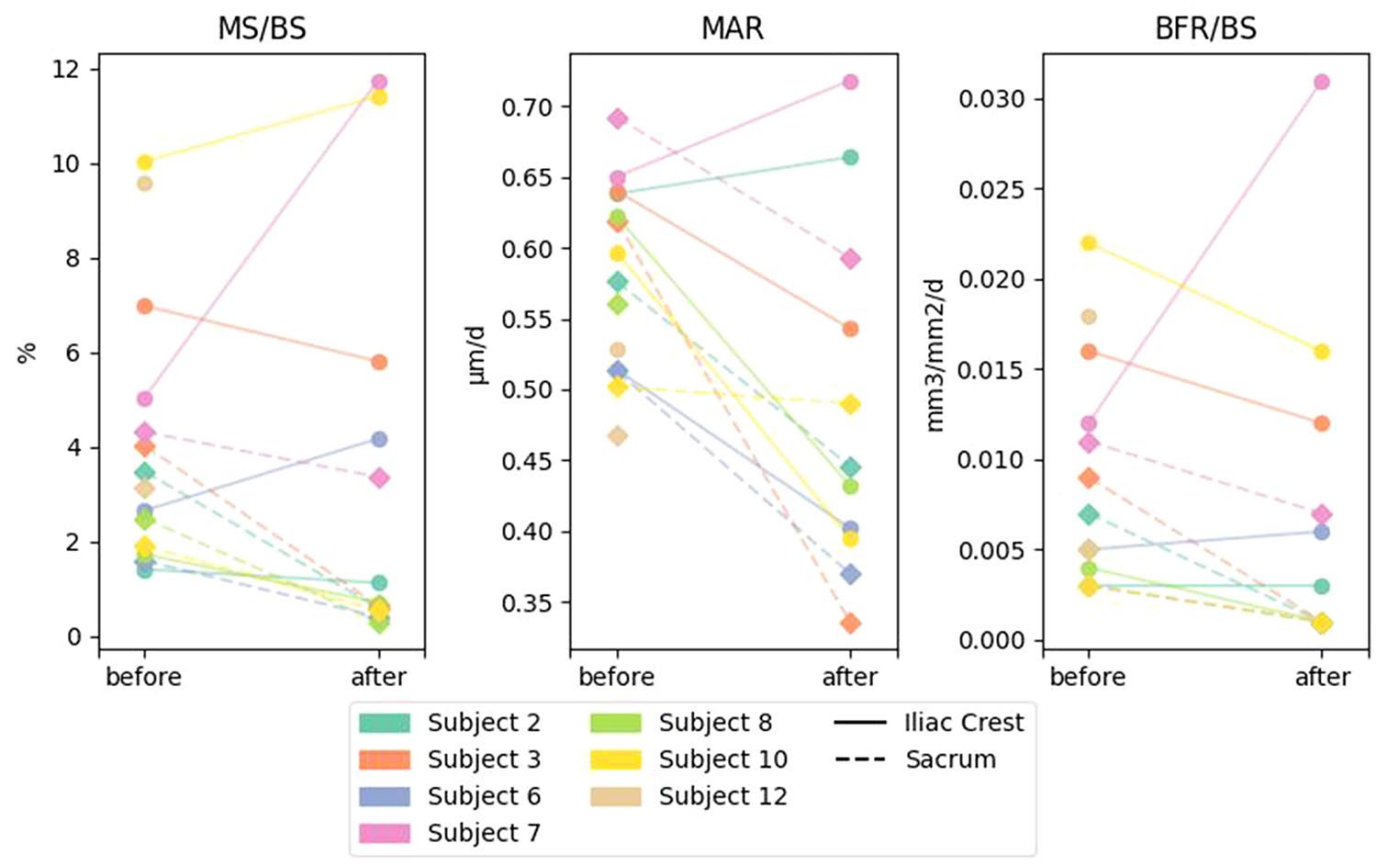
A graph showing the mineralizing surface (MS/BS), mineral apposition rate (MAR) and bone formation rate (BFR/BS) in the cancellous envelope before and after RT in the sacrum and iliac crest

**Fig. 5:**
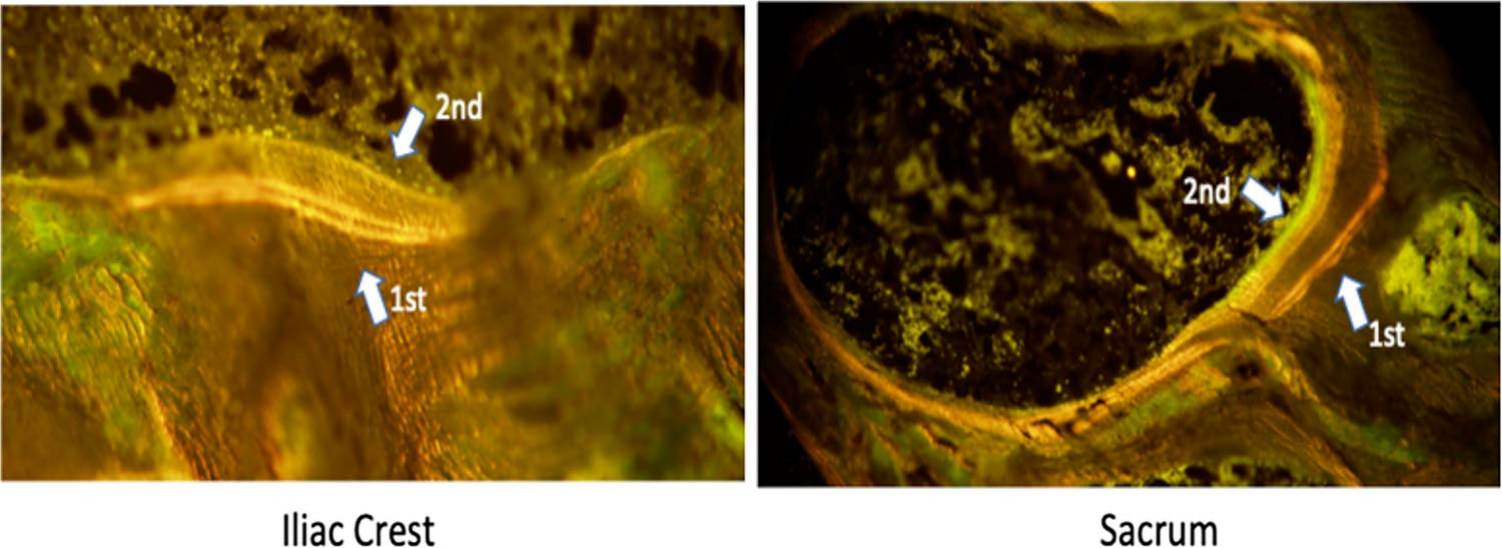
Representative images of cancellous bone in the iliac crest and sacrum of the same patients showing first and second set of tetracycline labels. Note the reduced extent of the second label in the sacrum following RT

The histomorphometric data are summarized in Tables 2 and 3. Table 2 shows the effect of radiation treatment on the dynamic parameters of bone formation. In the non-irradiated site (iliac crest) the bone formation parameters were unchanged following treatment in all four bone envelopes, whereas in the irradiated site (sacrum), there were significant decreases in single-labeled surface (sLS/BS), double-labeled surface (dLS/BS), mineralizing surface (MS/BS), mineral apposition rate (MAR) and bone formation rate (BFR/BS) in the cancellous envelope. Figure 4 shows these last three parameters in the cancellous envelope in each patient before and after RT in the sacrum and iliac crest. Similar trends were seen in the endocortical and intracortical envelopes. Figure 5 shows representative images of the tetracycline labels in the cancellous envelope of iliac crest and sacrum of the same patient. The effects on the extent of labeled surfaces (sLS/BS, dLS/BS and MS/BS) were quantitatively greater than those on MAR. There was hardly any formation occurring in the periosteal envelope of both the iliac crest and sacrum prior to radiation treatment and therefore no change was seen with treatment.

**Table 2:** Dynamic parameters of bone formation in four bone envelopes before and after radiation treatment in the iliac crest (non-irradiated site) and sacrum (irradiated site)

Parameter	Site	Before	After	n
Cancellous envelope				
sLS/BS (%)	Iliac Crest	5.65 (2.04, 8.34)	5.98 (1.11, 10.75)	7
	Sacrum	3.15 (2.17, 4.06)	0.90 (0.61, 1.01)*	7
dLS/BS (%)	Iliac crest	2.22 (0.71, 3.53)	1.48 (1.00, 2.57)	6
	Sacrum	1.13 (0.82, 2.21)	0.14 (0.11, 0.20)*	6
MS/BS (%)	Iliac crest	5.05 (1.73, 9.58)	4.99 (1.13, 11.41)	6
	Sacrum	3.15 (1.91, 4.03)	0.58 (0.41, 0.64)*	6
MAR (µm/day)	Iliac crest	0.62 (0.53, 0.64)	0.49 (0.40, 0.66)	6
	Sacrum	0.56 (0.50, 0.62)	0.45 (0.37, 0.50)*	5
BFR/BS (mm^3^/mm^2^/day)	Iliac crest	0.012 (0.004, 0.018)	0.009 (0.003, 0.016)	6
	Sacrum	0.005 (0.003, 0.009)	0.001 (0.001, 0.001)*	5
Aj.AR (µm/day)	Iliac Crest	–	0.27 (0.15, 0.48)	6
	Sacrum	–	0.07 (0.03, 0.18)	5
MLT (days)	Iliac crest	–	23.34 (16.28, 40.77)	6
	Sacrum	–	68.10 (34.10, 110.51)	5
OMT (days)	Iliac crest	–	11.60 (8.36, 14.07)	6
	Sacrum	–	10.13 (9.69, 10.30)	5
Act.F (cycle/year)	Iliac crest	–	0.33 (0.12, 0.60)	6
	Sacrum	–	0.04 (0.04, 0.05)	5
Endocortical envelope				
sLS/BS (%)	Iliac crest	6.66 (4.39, 10.87)	6.56 (4.21, 10.98)	7 (6)
	Sacrum	4.00 (3.26, 6.74)	2.20 (1.12, 10.41)	5 (4)
dLS/BS (%)	Iliac crest	5.37 (2.59, 8.68)	3.16 (1.59, 7.04)	7 (6)
	Sacrum	1.45 (1.16, 7.55)	0.21 (0.00, 1.25)	5 (4)
MS/BS (%)	Iliac crest	9.99 (6.75, 14.21)	5.83 (4.92, 15.58)	7 (6)
	Sacrum	4.82 (2.79, 9.55)	2.35 (1.29, 5.72)	5 (4)
MAR (µm/day)	Iliac crest	0.51 (0.48, 0.53)	0.44 (0.41, 0.48)	6 (6)
	Sacrum	0.50 (0.44, 0.55)	0.47 (0.19, 0.76)	5 (2)
BFR/BS (mm^3^/mm^2^/day)	Iliac crest	0.020 (0.016, 0.022)	0.011 (0.007, 0.024	6 (6)
	Sacrum	0.010 (0.004, 0.017)	0.004 (0.001, 0.007)	5 (2)
Aj.AR (µm/day)	Iliac crest	–	0.35 (0.24, 0.39)	6
	Sacrum	–	0.12 (0.07, 0.18)	2
MLT (day)	Iliac crest	–	15.48 (13.31, 34.23)	6
	Sacrum	–	38.73 (37.18, 40.28)	2
OMT (day)	Iliac crest	–	13.91 (11.41, 16.31	6
	Sacrum	–	11.95 (8.58, 15.31)	2
Act.F (cycle/year)	Iliac crest	–	0.43 (0.25, 0.79)	6
	Sacrum	–	0.16 (0.06, 0.26	2
Intracortical envelope				
sLS/BS (%)	Iliac crest	8.17 (7.84, 14.06)	15.10 (7.85, 20.19)	7 (6)
	Sacrum	7.96 (0.85, 8.74)	0.35 (0.00, 1.12)	5 (4)
dLS/BS (%)	Iliac crest	3.76 (1.36, 9.19)	4.40 (0.91, 9.15)	7 (6)
	Sacrum	2.04 (0.73, 8.88)	0.00 (0.00, 0.30)	5 (4)
MS/BS (%)	Iliac crest	7.03 (5.38, 13.11)	14.02 (8.26, 16.44)	7 (6)
	Sacrum	6.02 (1.15, 13.25)	0.38 (0.00, 0.86)	5 (4)
MAR (µm/day)	Iliac crest	0.96 (0.64, 1.20)	0.53 (0.50, 0.59)	7 (5)
	Sacrum	0.76 (0.53, 1.12)	0.68	4 (1)
BFR/BS (mm^3^/mm^2^/day)	Iliac crest	0.029 (0.017, 0.047)	0.032 (0.032, 0.035	7 (5)
	Sacrum	0.013 (0.004, 0.053)	0.000 (0.000, 0.002)	5 (3)
Aj.AR (µm/day)	Iliac crest	–	0.44 (0.43, 0.45)	5
	Sacrum	–	0.03 (0.00, 0.07)	2
MLT (day)	Iliac crest	–	16.36 (12.56, 20.33)	5
	Sacrum	–	87.15	1
OMT (day)	Iliac crest	–	14.29 (12.22, 15.10)	5
	Sacrum	–	8.45	1
Act.F (cycle/year)	Iliac crest	–	0.87 (0.83, 0.92)	5
	Sacrum	–	0.00 (0.00, 0.08)	3
Periosteal envelope				
sLS/BS (%)	Iliac crest	0.00 (0.00, 0.77)	0.00 (0.00, 0.00)	7 (6)
	Sacrum	0.00 (0.00, 0.00)	0.00 (0.00, 0.00)	5 (4)
dLS/BS (%)	Iliac crest	0.00 (0.00, 0.00)	0.00 (0.00, 0.00)	7 (6)
	Sacrum	0.00 (0.00, 0.00)	0.00 (0.00, 0.00)	5 (4)
MS/BS (%)	Iliac crest	0.00 (0.00, 0.38)	0.00 (0.00, 0.00)	7 (6)
	Sacrum	0.00 (0.00, 0.00)	0.00 (0.00, 0.00)	5 (5)
MAR (µm/day)	Iliac crest	0.33	0.47	1 (1)
	Sacrum	1.50	0.98	1 (1)
BFR/BS (mm^3^/mm^2^/day)	Iliac crest	0.00 (0.00, 0.00)	0.00 (0.00, 0.00)	6 (6)
	Sacrum	0.00 (0.00, 0.00)	0.00 (0.00, 0.002)	5 (4)
Aj.AR (µm/day)	Iliac crest	–	–	
	Sacrum	–	–	
MLT (day)	Iliac crest	–	–	
	Sacrum	–	–	
OMT (day)	Iliac crest	–	–	
	Sacrum	–	–	
Act.F (cycle/year)	Iliac crest	–	–	
	Sacrum	–	–	

Data are expressed as medians and inter-quartile ranges. Significant differences are marked with an asterisk (*)

*sLS* single-labeled surface, *BS* bone surface, *dLS* double-labeled surface, *MS* mineralizing surface, *MAR* mineral apposition rate, *BFR* bone formation rate, *Aj.AR* adjusted apposition rate, *MLT* mineralization lag time, *OMT* osteoid maturation time, *Act.F* activation frequency, *n* number of subjects before, *n ()* number of subjects after

**Table 3:** Static histomorphometric parameters in four bone envelopes after radiation treatment in the iliac crest (non-irradiated site) and sacrum (irradiated site)

Parameter	Site	After	n
Cancellous envelope			
OV/BV (%)	Iliac crest	1.61 (0.59, 2.44	7
	Sacrum	0.83 (0.48, 1.14)	7
OS/BS (%)*	Iliac crest	10.29 (5.50, 17.45)	7
	Sacrum	4.66 (1.59, 8.48)	7
O.Th (µm)	Iliac crest	6.66 (5.55, 7.86)	7
	Sacrum	4.46 (3.58, 4.52)	7
W.Th (µm)	Iliac crest	26.15 (24.44, 27.10)	7
	Sacrum	24.33 (22.51, 25.53)	7
Ob.N/BS (%)*	Iliac crest	0.897 (0.162, 1.247)	7
	Sacrum	0.052 (0.007, 0.189)	7
Ob.S/BS (%)*	Iliac crest	1.628 (0.259, 2.384)	7
	Sacrum	0.104 (0.009, 0.288)	7
ES/BS (%)*	Iliac crest	6.42 (4.46, 8.36)	7
	Sacrum	3.45 (2.70, 7.04)	7
Oc.N/BS (%)	Iliac crest	0.029 (0.019, 0.084)	7
	Sacrum	0.009 (0.004, 0.068)	7
Oc.S/BS (%)	Iliac crest	0.124 (0.069, 0.437)	7
	Sacrum	0.035 (0.011, 0.294)	7
Endocortical envelope			
OS/BS (%)	Iliac crest	11.87 (4.41, 18.16)	7
	Sacrum	11.47 (5.45, 13.74)	5
O.Th (µm)	Iliac crest	6.04 (4.96, 7.89)	7
	Sacrum	5.38 (3.64, 6.50)	5
W.Th (µm)	Iliac crest	29.65 (26.97, 30.18)	7
	Sacrum	27.40 (27.25, 28.25)	5
Ob.N/BS (%)	Iliac crest	2.285 (0.947, 3.430)	7
	Sacrum	0.339 (0.226, 0.517)	5
Ob.S/BS (%)	Iliac crest	2.777 (2.579, 8.682)	7
	Sacrum	0.451 (0.226, 0.723)	5
ES/BS (%)	Iliac crest	5.79 (3.95, 9.34)	7
	Sacrum	5.22 (4.97, 6.23)	5
Oc.N/BS (%)	Iliac crest	0.108 (0.067, 0.355)	7
	Sacrum	0.000 (0.000, 0.197)	5
Oc.S/BS (%)	Iliac crest	0.643 (0.267, 1.299)	7
	Sacrum	0.000 (0.000, 0.919)	5
Intracortical envelope			
OS/BS (%)	Iliac crest	19.54 (8.48, 25.23)	7
	Sacrum	5.61 (1.92, 8.67)	5
O.Th (µm)	Iliac crest	7.26 (5.41, 9.22)	7
	Sacrum	4.71 (3.41, 5.07)	5
W.Th (µm)*	Iliac crest	39.64 (36.10, 45.02)	7
	Sacrum	31.37 (29.77, 32.03)	5
Ob.N/BS (%)	Iliac crest	1.355 (0.259, 5.273)	7
	Sacrum	0.000 (0.000, 0.476)	5
Ob.S/BS (%)	Iliac crest	2.970 (0.427, 9.809)	7
	Sacrum	0.000 (0.000, 2.376)	5
ES/BS (%)	Iliac crest	5.19 (1.34, 9.90)	7
	Sacrum	10.18 (3.97, 11.81)	5
Oc.N/BS (%)	Iliac crest	0.122 (0.000, 0.261)	7
	Sacrum	0.014 (0.000, 0.826)	5
Oc.S/BS (%)	Iliac crest	0.625 (0.000, 0.966)	7
	Sacrum	0.070 (0.000, 2.684)	5

Data are expressed as medians and inter-quartile ranges. Significant differences are marked with an asterisk (*).Variables with a “–”can only be assessed in the single biopsy obtained after treatment

*OV* osteoid volume, *BV* bone volume, *OS* osteoid surface, *BS* bone surface, *O.th* osteoid thickness, *W.Th* wall thickness, *Ob.N* osteoblast number, *Ob.S* osteoblast surface, *ES* eroded surface, *Oc.N* osteoclast number, *Oc.S* osteoclast surface

When comparing the sacrum with the iliac crest iliac crest after treatment (Tables 2, 3), the results were consistent with the decrease in bone formation with radiation treatment of the sacrum. Thus, adjusted apposition rate (Aj.AR), and activation frequency (Act.F) were generally lower in the sacrum than in the iliac crest and the mineralization lag-time was prolonged (Table 2). Moreover, osteoid volume (OV/BV), osteoid surface (OS/BS), osteoblast number (Ob.N/BS), osteoblast surface (Ob.S,/BS), eroded surface (ES/BS), osteoclast number (Oc.N/BS), and osteoclast surface (Oc.S/BS) were generally all lower in the sacrum than the iliac crest, consistent with reductions in BFR and Act.F (Table 3).

## Discussion

### Key Results

High-dose radiation is increasingly used for the (adjuvant) treatment of malignancies, such as malignant sacral tumors. Despite the beneficial effects of radiation, detrimental effects such as insufficiency fractures have been reported [5]. In this prospective study we assessed the effects of high-dose radiation on bone using QCT and bone histomorphometry. We observed a decrease in volumetric BMD in the irradiated bone compared to the non-irradiated bone, immediately after radiation. Furthermore, using quadruple tetracycline labeling, we demonstrated a marked decrease in the bone formation rate in the sacrum following radiation treatment. The reduction in bone formation rate was due to a decrease in the extent of forming surfaces (MS/BS) as well as a decrease in the linear rate of matrix apposition (MAR) at each formation site, although the former effect was more prominent. Comparison of the static histomorphometric parameters between the sacrum and the iliac crest confirmed that the decrease in bone formation was due to a decrease in activation frequency with marked reductions in osteoclast and osteoblast number, presumably due to a toxic effect of the radiation on both these cell types.

### Limitations

Some limitations need to be taken into consideration when interpreting these results. First, due to the low incidence of sacral tumors, our study cohort was small. Because of this, we were for instance not able to control for covariates that may impact BMD. Second, for pragmatic reasons we chose to compare bone histomorphometry variables of the irradiated sacrum to the non-irradiated iliac crest. We believe one of the strengths of the study is the fact that we compare two sites within each subject. However, we do not know if the untreated values in the two sites are similar. Nevertheless, for our primary endpoint (BFR by quadruple labeling) we have baseline and post-radiation measurements in each subject, which revealed a marked decrease in BFR. Even though we do not have untreated values for the sacrum, the marked lower values for the static parameters of formation and resorption in the sacrum compared to the iliac crest are consistent with longitudinal reduction in BFR in the sacrum. Third, not all patients received the same amount of proton–photon RT. They did, however, receive the same preoperative radiation dose of 50.4 Gy which has been our institutional standard for sacral malignant tumors, and similar total dose of radiation. Fourth, the timing of treatment and study points differed among patients depending upon the dates of their prescheduled clinic visits. Finally, in this study we only looked at the short term effect of high-dose radiation on bone. Future studies are necessary to determine whether these effects are still of significance in the long term.

The mechanisms through which radiation leads to bone fragility are mostly known through animal studies. Bone is a dynamic tissue which remodels constantly to maintain its functions. Bone-lining cells, osteocytes, osteoblasts and osteoclasts are the key players in this remodeling process and a tight balance between bone resorption by osteoclasts and bone formation by osteoblasts is needed to ensure bone quality [20]. The exact effect of RT on these different cell types may depend on type of RT, total dose, dose rate, field size and other RT specific parameters [21]. Generally, an early activation of osteoclasts is thought to lead to an increase in bone resorption and therefor a decrease in trabecular bone volume. This sudden increase is followed by a decrease of osteoclasts, leading to a long-term depletion [22]. This may be attributed to the fact that osteoclast progenitors are located in the bone marrow and known to be radiosensitive [21, 23–25].

### Bone Mineral Density

In a recent study our group retrospectively analyzed CTs of 21 patients with a sacral tumor before and after RT to assess the effects of high-dose rt on trabecular BMD using CT attenuation measurements [14]. A decrease in trabecular BMD was observed in the irradiated lumbar vertebrae but not in the non-irradiated lumbar vertebrae. In the current study, we confirmed these findings. Similar results were found in a study of 42 patients with a locally advanced abdominal malignancy treated with chemoradiation [26]. The authors observed a reduction in BMD in the thoracic and lumbar spine after RT. However, another study consisting of 19 patients treated with a combination of surgery and RT for soft tissue sarcoma of the extremity found no decrease in BMD RT within long bones [27]. This may be due to the greater proportion of cortical bone within long bones, which is affected less than trabecular bone by RT.

As described in animal studies, BMD is thought to decrease first due to increased resorption of trabecular bone and then later increase due to a long-term depletion of osteoclasts [22]. A study of 60 patients treated with fractionated intensity-modulated radiation therapy (IMRT) or three-dimensional conformal radiation therapy (3DCRT) for palliative management of spinal metastases found an increase in BMD after 3 months [28]. Our study subjects may not be comparable to this study [28] due to a difference in total radiation dose, but it would be interesting to see if there would also be an increase in BMD in the long run.

## Conclusion

This pilot study shows a decrease of BMD and bone formation rate after high-dose RT in patients treated for a sacral malignant tumor. Further studies are needed to more fully characterize the long-term and systemic effects of RT on bone.

## Declarations

### Location

This research was performed at the Massachusetts General Hospital in Boston, MA.
